# Hürthle Cells on Fine-Needle Aspiration Cytology Are Important for Risk Assessment of Focally PET/CT FDG Avid Thyroid Nodules

**DOI:** 10.3390/cancers12123544

**Published:** 2020-11-27

**Authors:** David N. Poller, Hakim Megadmi, Matthew J. A. Ward, Pierpaolo Trimboli

**Affiliations:** 1 Departments of Cytology & Pathology, Queen Alexandra Hospital, Portsmouth PO6 3LY, UK; 2 Department of Nuclear Medicine, Queen Alexandra Hospital, Portsmouth PO6 3LY, UK; hakim.megadmi@porthosp.nhs.uk; 3 Department of ENT Surgery, Queen Alexandra Hospital, Portsmouth PO6 3LY, UK; matthew.ward@porthosp.nhs.uk; 4 Clinic of Endocrinology, Ente Ospedaliero Cantonale, 6900 Lugano, Switzerland; Pierpaolo.Trimboli@eoc.ch; 5 Faculty of Biomedical Sciences, Università della Svizzera Italiana (USI), 6900 Lugano, Switzerland

**Keywords:** FDG PET/CT, thyroid, cytology, Hürthle cell, malignancy, risk

## Abstract

**Simple Summary:**

PET/CT fluorodeoxyglucose (FDG) scans are routinely used in patients to detect signs of malignant tumours or evidence of inflammation in the body. A total of 1–2% of patients show focal thyroid gland FDG uptake and 35–40% are malignant. FDG also detects metabolically active lesions containing mitochondria, known as Hürthle cells. Over 3 years, 47 patients in one hospital were found to have focal thyroid gland uptake. A total of 18 (38.2%) of the patients had malignancy, 15 (31.9%) had benign lesions that contained Hürthle cells and 14 (29.8%) had focally increased thyroid gland FDG PET/CT uptake with no cause identified. Exclusion of the Hürthle cell patients increased the risk of malignancy of the remaining PET-positive nodules from 38% to 68%. It is important to recognize Hürthle cells on FNA cytology in FDG PET/CT-positive nodules as this affects the risk of malignancy and the clinical management of focally FDG PET/CT-positive nodules.

**Abstract:**

This study assesses the role of [^18^F] FDG PET/CT, fine needle aspiration (FNA) cytology and ultrasound in the 1–2% of patients with focally positive thyroid nodules on FDG PET/CT. All FDG PET/CT scans with focally increased thyroid FDG PET/CT uptake performed over 37 months in one institution were matched to patients undergoing thyroid FNA. Diffuse FDG PET/CT uptake patients were excluded. A total of 47 patients showed focally increased thyroid uptake. Consistent with previous studies, 18 (38.2%) patients had malignancy—12 primary thyroid carcinoma, 1 parathyroid carcinoma, 3 metastatic carcinoma to the thyroid and 2 lymphoma. A total of 15 (31.9%) lesions categorized as non-malignant contained Hürthle cells/oncocytes. A total of 14 lesions (29.8%) had focally increased FDG PET/CT uptake with no specific cytological or histopathological cause identified. No focally PET avid Hürthle cell/oncocytic lesions were found to be malignant. Exclusion of oncocytic lesions increased the calculated risk of malignancy (ROM) of focally PET avid nodules from 38% to 68%. It may be useful to exclude focally FDG PET/CT avid Hürthle cell/oncocytic lesions, typically reported as follicular neoplasm or suspicious for a follicular neoplasm, Hürthle cell type (Oncocytic) type, RCPath Thy 3F: Bethesda IV or sometimes Thy 3a: Bethesda III FNAs) from ROM calculations. Oncocytic focally PET/CT FDG avid lesions appear of comparatively lower risk of malignancy and require investigation or operation but these lesions should be readily identified by FNA cytology on diagnostic work up of focally PET avid thyroid nodules.

## 1. Introduction

Positron emission tomography (known as PET) using [^18^F]-2-fluoro-2-deoxy-D-glucucose (also known as ^18^F-FDG) is diagnostically useful, as cancer cells [1] and inflammatory lesions characteristically show increased ^18^F-FDG uptake due to increased rates of anaerobic glycolysis. ^18^F-FDG glucose is metabolized via glycolysis to ^18^FDG-6-phosphate but it cannot be further metabolized intracellularly via glycolysis, so it accumulates in cells and tissues if there is increased tissue uptake. ^18^F-FDG (referred to as FDG later throughout this article) PET is commonly performed in conjunction with computerized tomography (CT). FDG PET/CT is often performed as a routine investigation for malignancy [2]. The uptake of FDG in the thyroid gland may be either focal or diffuse. The causes of diffuse uptake include thyrotoxicosis, thyroiditis including Hashimoto’s thyroiditis, and other inflammatory lesions such as abscesses [3]. Increased FDG uptake is seen in metabolically active cells. Increased metabolic activity due to large numbers of mitochondria is also present in Hürthle cell/oncocytic thyroid lesions [4,5,6] and other head and neck tumours, such as in Warthin-type salivary gland tumours [7]. Focally FDG PET/CT avid incidental thyroid lesions 1 cm or greater in size require further investigation because of an increased risk of malignancy [8,9,10,11]. Investigation is commonly performed via ultrasound and fine-needle aspiration (FNA), unless disseminated disease is identified and the prognosis of an alternative malignancy precludes further investigation [12]. The stated risk of malignancy in FDG PET/CT focally avid thyroid nodules is approximately 35–40%, although this varies quite widely depending on the case series and the patient cohort characteristics [11,13]. In some centres, FDG PET/CT is used as part of the primary diagnostic work up for cytologically indeterminate thyroid nodules [13,14] but FDG PET/CT is not utilized in our centre for diagnostic assessment of cytologically indeterminate thyroid nodules. All focally FDG PET/CT avid nodules in our series were detected during routine investigations for either cancers at other sites, assessment of possible recurrence of thyroid carcinoma, or assessment of benign inflammatory conditions. The aim of this study was to assess the role of cytology in the risk assessment of PET/CT focally avid thyroid lesions.

## 2. Results

A total of 47 patients were identified with focally FDG PET/CT avid thyroid nodules in the 37 month period January 2017–February 2020. Eighteen patients had a final diagnosis of malignancy. The clinical characteristics, FDG PET/CT results, nodule size on ultrasound, British Thyroid Association (BTA) ‘U’ class, ‘Thy’ category, cytology diagnosis and histology diagnoses (if available) are shown in Table S1. Receiver operating curves were then constructed for maximum standardized uptake value (SUV*max*) for the cases with a final malignant diagnosis (Table S1) cf. oncocytic/Hürthle cell lesions (Table S2) cf. other lesions (Table S3). Eighteen patients had a final diagnosis of malignancy. The clinical characteristics, FDG PET/CT results, nodule size on ultrasound, BTA ‘U’ class, ‘Thy’ category, cytology diagnosis and histology diagnoses (if available) are shown in Table S1. There were 5 male and 13 female patients—mean age, 62.1, range 37–80. Seven cases were reported as Thy 5, equivalent to The Bethesda System for Reporting Thyroid Cytopathology (TBS) category I; five cases were Thy 4, equivalent to TBS category V; four cases were Thy 3F, equivalent to TBS category IV; one case was Thy 3a, equivalent to TBS category III; and one case was Thy 1, equivalent to TBS category I [15,16]. None of these cases showed Hürthle cells/oncocytes. The 18 cases with a final diagnosis of malignancy comprised 8 primary thyroid papillary thyroid carcinomas, 2 follicular thyroid carcinomas, 2 medullary thyroid carcinomas, 1 parathyroid carcinoma, 1 Hodgkin’s lymphoma, 1 follicular lymphoma grade 3B, 1 anal adenocarcinoma metastatic to the thyroid, 1 pancreatic adenocarcinoma metastatic to the thyroid and 1 squamous cell carcinoma of the bronchus confirmed on bronchial biopsy. SUV*max* data was recorded for 17 of 18 cases—mean 14.9 (range 2.7–63.2, median 8.2). 

**Figure 1:**
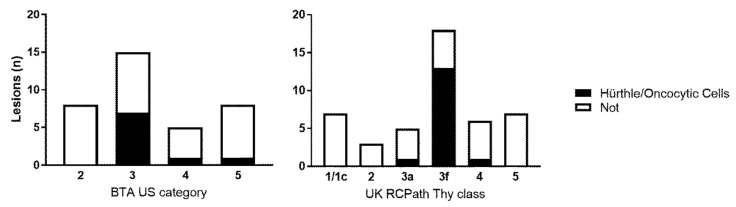
Results for all 47 focally FDG PET/CT positive patients by British Thyroid Association ‘U’ Category (**left**) and UK RCPath ‘Thy’ class (**right**). Hürthle cell cases are shaded black.

Fifteen patients had Hürthle cells/oncocytes evident on FNA cytology but subsequent investigations failed to confirm evidence of a malignant lesion. There were 4 males, and 11 females—mean age 67.3, range 46–88. The clinical characteristics, FDG PET/CT results, nodule size on ultrasound, BTA ‘U’ class, ‘Thy’ category, cytology diagnosis and histology diagnosis (if available) are shown in Table S2. All these cases with two exceptions were reported as Thy 3F (neoplasm possible follicular lesion) which in TBS is equivalent to TBS category IV [15,16]. One case was reported as Thy 3a, equivalent to TBS category III; and one case as Thy 4, equivalent to TBS category V [15,16]. Five of the 15 patients had histological assessment. One case was an infarcted Hürthle cell follicular adenoma (case 1), a second was a 11 mm-sized follicular adenoma with variable oncocytic change (case 2), a third was a non-invasive follicular thyroid neoplasm with papillary like nuclei (NIFTP) but with background oncocytic change (case 3), a fourth case (case 5) showed multiple benign adenomatoid nodules focally with oncocytic appearances and a fifth case showed an oncocytic follicular adenoma (case 14). SUV*max* data was recorded for all 15 cases—mean 10.6 (range 2.9–30, median 7.3). The majority of the nodules containing Hürthle/oncocytic cells were in the indeterminate cytological or ultrasound classes (i.e., Thy 3F: equivalent to TBS IV and BTA ultrasound class U3) (Figure 1).

Fourteen patients had focally FDG PET/CT avid thyroid nodules without evidence of either thyroid malignancy or evidence of Hürthle/oncocytic cells on cytology. There were 4 male and 10 female patients—mean age 68.6, range 44–87. The clinical characteristics, FDG PET/CT results, nodule size on ultrasound, BTA ‘U’ class, ‘Thy’ category, cytology diagnosis and histology diagnosis (if available) are shown in Table S3. One case was reported as Thy 3F (neoplasm possible follicular lesion), equivalent to TBS category IV; three cases as Thy 3a, equivalent to TBS category III, three cases as Thy 2, equivalent to TBS category II; and seven cases as Thy 1 or Thy 1c, equivalent to TBS category I [15,16]. SUV*max* data was recorded for all 14 cases—mean 4.6 (range 2.2–7.6, median 4.6).

**Figure 2:**
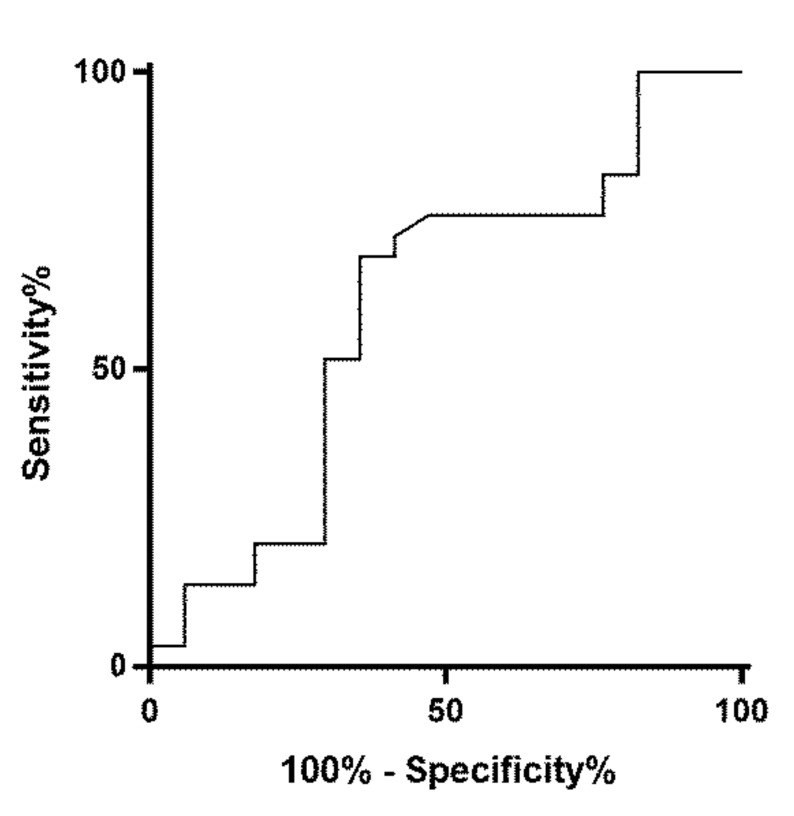
ROC curve analysis: SUV*max* all malignant FDG PET/CT focally avid cases vs. all Hürthle/oncocytic lesions and all other cases. Area under ROC curve by extended trapezoidal rule = 0.605477. Wilcoxon estimate of area under ROC curve = 0.605477. DeLong standard error = 0.093527: 95% CI = 0.422167 to 0.788786. Optimum cut-off point selected = 6.1.

**Figure 3:**
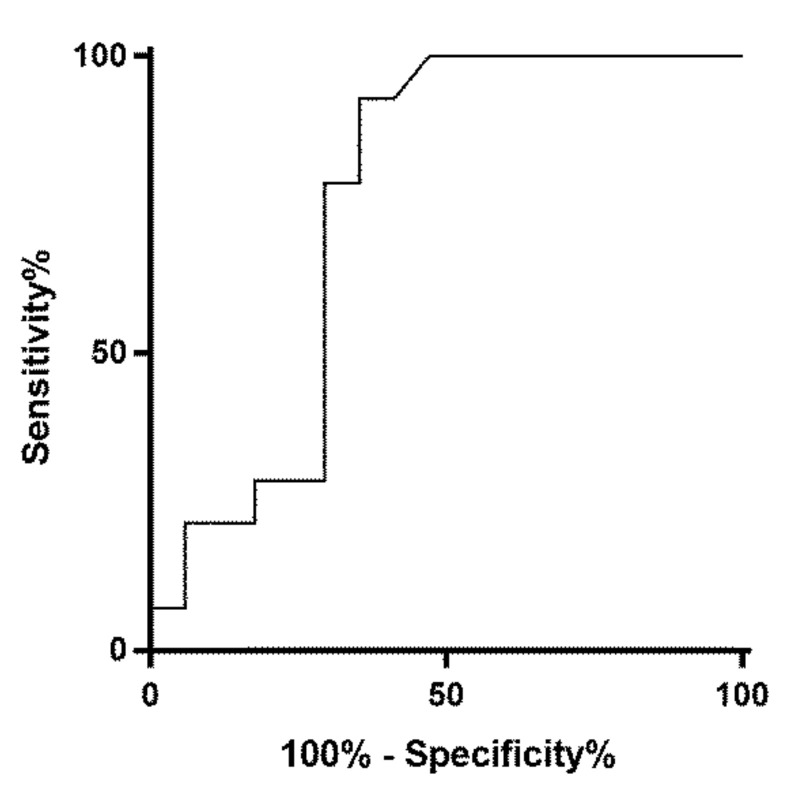
ROC curve analysis: SUV*max* all malignant FDG PET/CT focally avid cases vs. all other cases excluding all Hürthle/oncocytic lesions. Area under ROC curve by extended trapezoidal rule = 0.75. Wilcoxon estimate of area under ROC curve = 0.75. DeLong standard error = 0.094412: 95% CI = 0.564955 to 0.935045. Optimum cut-off point selected = 6.1.

The area under the receiver operating curve for all cases with a confirmed final diagnosis of malignancy (Table S1) cf. all cases of oncocytic/Hürthle cell lesions plus other cases without a final diagnosis of malignancy (Tables S2 and S3) was 0.605 with an optimum SUV*max* cut off of 6.1 (Figure 2). The area under the receiver operating curve for all cases with a confirmed diagnosis of malignancy (Table S1) cf. all other cases without a final diagnosis of malignancy (Table S3) if oncocytic/Hürthle cell lesions were excluded was 0.75 with an optimum SUV*max* cut off of 6.1 (Figure 3).

## 3. Discussion

Thyroid FNA is useful for triage of focally FDG avid PET/CT thyroid nodules. The results of FNA cytology of focally FDG PET/CT avid thyroid lesions should be considered in conjunction with the clinical history, other clinical investigations and the ultrasound characteristics [12]. The role of this service evaluation was not to produce any changes in treatment, care, or services and the study outcomes are consistent with multiple previous studies in the published literature. These results show that the optimal diagnostic method for categorisation of benign vs. malignant focally FDG PET-positive nodules is, as expected, either FNA cytology or the ultrasound characteristics while the FDG PET/CT uptake as assessed by SUV*max* is less predictive of a final diagnosis of malignancy in our patient cohort as compared to FNA cytology or the ultrasound characteristics of the nodule. 

In our study, approximately one-third (31.9%) of thyroid nodules which were focally FDG PET/CT avid contained Hürthle cells/oncocytes as the presumed cause of the increased FDG uptake—these are shown in Table S2—while 31.9% of the cases did not have a clearly identifiable cause for increased focal FDG PET/CT uptake as these were neither malignant nor contained Hürthle cells/oncocytes and no specific reason for increased FDG uptake was identified (Table S3). While it is well known that oncocytic thyroid lesions, both benign and malignant, typically have increased FDG PET/CT uptake, many published studies of thyroid FDG PET/CT do not consider oncocytic thyroid lesions as a separate entity. In most published studies oncocytic/Hürthle cell cases are included in SUV*max*, sensitivity, and specificity analyses as either ‘*benign*’ lesions or as ‘*malignant*’ lesions, rather than as an oncocytic lesion/Hürthle cell intermediate risk of malignancy subgroup. Studies that have taken this latter approach to oncocytic/Hürthle lesions of the thyroid have produced similar results to ourselves. Pathak et al. showed an overall accuracy of FDG PET/CT of 56% but the area under the SUV*max* receiver operating curve for benign and non-malignant oncocytic neoplasms was 0.79 (95% CI 0.64–0.94); *p* = 0.001 with a SUV*max* optimal cut off of 3.25 and an overall increased accuracy of 81% if the eight oncocytic lesions, all of which were benign adenomas, were excluded from SUV*max* calculations with an optimum SUV*max* of 3.25 [17]. Munoz-Perez et al. also adopted a similar approach to oncocytic thyroid lesions, calculating SUV*max* of oncocytic lesions separately from other non-oncocytic/Hürthle cell lesions [18]. 

Oncocytic thyroid neoplasms, also known as Hürthle cell neoplasms, are defined as tumours with more than 75% oncocytic cells and are classified as oncocytic adenoma and oncocytic carcinoma. Lesions with less than 75% oncocytic cells are classified as lesions with ‘oncocytic/Hürthle cell features’ [19]. Oncocytic lesions of the thyroid on FNA cytology are usually relatively straightforward to diagnose, the diagnostic pitfalls for benign oncocytic lesions include most commonly Hashimoto’s thyroiditis, multinodular goiter, and less commonly Graves’ disease, or following neck irradiation with radioiodine or after partial thyroidectomy [20,21]. Non-thyroid neoplastic or malignant head and neck lesions that contain oncocytic cells which may fall in the cytological differential diagnosis of FDG PET avid head and neck lesions include Warthin tumour, oncocytoma of salivary glands and parathyroid lesions. Other primary thyroid oncocytic tumours include oncocytic variant of papillary thyroid carcinoma and oncocytic variant of medullary thyroid carcinoma [22].

This study suggests that focally FDG PET/CT avid thyroid nodules and high-risk FNA findings, in this study classified cytologically as UK RCPath Thy 4 or Thy 5, equivalent to The Bethesda System for Reporting Thyroid Cytopathology (TBS) [15] categories V and VI can be managed clinically as high-risk thyroid lesions based on the combination of the clinical presentation, the ultrasound characteristics, and the cytological findings after multidisciplinary discussion [23]. In this admittedly small series of 18 cases with a final diagnosis of malignancy, only one case was reported as non-diagnostic, Thy 1, equivalent to TBS category I; one case was Thy 3a, neoplasm possible-atypia equivalent to TBS category III; and all the remaining cases were reported as either Thy 3F neoplasm possible, equivalent to TBS category IV, Thy 4 suspicious of malignancy equivalent to TBS category V, or malignant Thy 5, equivalent to TBS category VI [15,16].

Interestingly, none of the cases with confirmed malignancy in our series and shown in Table S1 contained Hürthle cells/oncocytes. While oncocytic malignant tumours of the thyroid do occur, oncocytic follicular carcinomas of the thyroid are comparatively rare. The majority of newly diagnosed thyroid carcinomas in modern series are papillary thyroid carcinomas, modern series reporting approximately 85% papillary thyroid carcinomas with follicular thyroid carcinomas comprising approximately 10–12%. Goffredo et al. in an analysis of SEER data from 1988 to 2009 found that oncocytic thyroid carcinomas comprised ~5% of all differentiated thyroid carcinomas, with oncocytic follicular carcinomas showing less of a female bias, and occurring in older patients relative to follicular thyroid carcinoma [24]. Published survival data for oncocytic thyroid carcinoma and follicular thyroid carcinoma also varies, and some series show that oncocytic thyroid carcinoma has worse prognosis that follicular thyroid carcinoma on long-term 10 year follow up, whereas other studies have not shown this [25,26,27,28,29,30].

The risk of malignancy of a Hürthle cell/oncocytic follicular thyroid neoplasm is in the region of approximately 35%, which corresponds to the expected risk of malignancy of a TBS category IV FNA [15,16], approximately equivalent to the risk of malignancy of a UK RCPath Thy 3F FNA [31] or TBS category IV [15]. Hence, Hürthle cell/oncocytic focally FDG PET/CT avid thyroid lesions in the absence of clinical characteristics or ultrasound characteristics pointing towards a diagnosis of malignancy according to current guidelines [8,15,32] can be managed as intermediate-risk lesions after clinical review or multidisciplinary discussion [23]. If comparatively lower risk of malignancy lesions containing Hürthle cells/oncocytes are excluded from ROM calculations, the ROM of focally FDG PET/CT avid thyroid nodules as shown in Tables S1 and S3 in this series was 68.1%. For cases such as those in Table S3 where there is no apparent cause identified for the increased focal FDG uptake, particularly if the cytology is benign or Thy 2, equivalent to TBS category II or Thy1, equivalent to TBS category I, it would, arguably, be reasonable after multidisciplinary discussion in these cases to undertake surveillance as these lesions are likely to be of low risk of malignancy.

## 4. Methods

The Queen Alexandra Hospital in Portsmouth, UK, is a major cancer referral centre located in the southern part of the United Kingdom, with a patient catchment of 650,000 plus, serving ~2% of the UK population. A list of all FDG PET/CT scans undertaken at The Queen Alexandra Hospital, Portsmouth, from 3rd January 2017 to 5th February 2020 was matched to a laboratory computer database of patient identifiers for patients also undergoing thyroid FNA cytology. This yielded 3961 scan results. Those patients that had both a FDG PET/CT scan and thyroid FNA cytology were the subject of this study. As this was a retrospective audit, patient information sheets were not offered and informed patient consent was not feasible to obtain. This service evaluation meets the criteria for clinical audit as per the host institution’s guidelines.

### 4.1. Patients and Clinical Characteristics

The search yielded 67 patients who had undergone a whole-body FDG PET/CT scan and ultrasound-guided thyroid FNA within a period of 6 months of a whole-body FDG PET/CT scan. A total of 20 patients showed either no increased FDG PET/CT uptake or diffuse FDG PET/CT uptake in the thyroid gland and these patients were excluded. The remaining 47 patients with focally increased thyroid FDG PET/CT uptake are the subject of this study. PET/CT FDG at the Queen Alexandra Hospital in Portsmouth FDG PET/CT is acquired using a Siemens Biograph TruePoint PET scanner with 40 slice CT, utilising integrated CARE dose 4D software. Patients were routinely fasted for six hours to ensure a blood glucose level within the recommended value of <12 mmol/L. Scanning was performed one hour post administration of 350 MBq [^18^F]-FDG and images were acquired for 2.5 min/bed position. The studies were reported by two specialized PET/CT consultants. Any incidental focal uptake within the thyroid was noted, with SUV*max* recorded and USS/FNA was advised. Almost all scans were reported by H.M. All FDG PET/CT-positive cases with increased uptake are routinely discussed at a multidisciplinary thyroid meeting. One author (DNP) systematically reviewed and retrieved the results of the FDG PET/CT scans from an electronic patient record, the results of the corresponding ultrasound scans, and correspondent cytology and histology of the thyroid where this was available.

Thyroid ultrasound (US) scans are performed by a team of radiologists in Portsmouth using Canon Aplio i800 ultrasound machines with Canon PLT-1005BT transducers. The results are reported descriptively as free text reports and are scored for US risk of malignancy using a British variant of TIRADS, The British Thyroid Association ‘U’ Classification for Thyroid FNA [12]. The British Thyroid Association ‘U’ classification has been validated in several studies [33,34,35,36].

FNA cytology. Thyroid aspirates were taken under ultrasound guidance in all cases in the ultrasound suite by the radiologist undertaking the FNA, typically 4 slides prepared, 2 stained with Giemsa and 2 stained with Papanicolaou, and then these were submitted to the cytology department where the majority were reported by DNP, the minority by other cytologists working in the Department of Pathology. Rapid on-site assessment is not utilized. The cytology results were reported using free text and given a score, classified using the UK RCPath ‘Thy’ terminology for reporting thyroid FNA cytology [32]. If a patient had more than one thyroid FNA, the highest Thy category was recorded in Tables S1–S3 and used for statistical analysis. This study was undertaken as a clinical audit and service evaluation, registration number 4823, Portsmouth Hospitals University NHS Trust, September 2020. As a clinical audit and service evaluation, this work does not require specific research ethical approval or institutional review board approval.

### 4.2. Statistics

The mean, median, and range of SUV*max* was calculated for the three groups of patients using StatsDirect statistical software, StatsDirect Ltd., Merseyside, UK. Patients were grouped by final diagnosis as (i) malignant FDG PET/CT avid thyroid lesions (Table S1), (ii) oncocytic/Hürthle cell lesion (Table S2) or (iii) other FDG PET/CT avid lesion—(lesions that were neither final diagnosis malignant or final diagnosis oncocytic/Hürthle cell lesions after investigation and multidisciplinary discussion) (Table S3). Receiver operating curves were then constructed for SUV*max* for the cases with a final malignant diagnosis cf. oncocytic/Hürthle cell lesions cf. other lesions.

## 5. Conclusions

Oncocytic/Hürthle thyroid lesions are common. Although oncocytic/Hürthle cell lesions are frequently FDG avid, oncocytic/Hürthle lesions are, in general, of relatively lower risk of malignancy. This service evaluation audit emphasizes the value of recognition of oncocytic/Hürthle thyroid lesions in thyroid FNA of focally FDG avid PET/CT thyroid nodules in the multidisciplinary assessment of patients with focally PET/CT avid thyroid nodules.

## Supplementary Materials

The following are available online at https://www.mdpi.com/2072-6694/12/12/3544/s1, Table S1: Malignant Cases: Patient Characteristics, PET/CT Findings, SUVmax, Nodule Size, British Thyroid Association ‘U’ Category, Thy Cytological Category, Cytological and Histopathological Diagnoses; Table S2: Hurthle Cell/Oncocytic Cases: Patient Characteristics, PET/CT Findings, SUVmax, Nodule Size, British Thyroid Association ‘U’ Category, Thy Cytological Category, Cytological and Histopathological Diagnoses; Table S3: Other Cases: Patient Characteristics, PET/CT Findings, SUVmax, Nodule Size, British Thyroid Association ‘U’ Category, Thy Cytological Category, Cytological and Histopathological Diagnoses.
