# Supplementary material for: Hürthle Cells on Fine-Needle Aspiration Cytology Are Important for Risk Assessment of Focally PET/CT FDG Avid Thyroid Nodules
Source: Cancers (Basel). 2020 Nov 27;12(12):3544. doi: 10.3390/cancers12123544 (PMC7761140; doi:10.3390/cancers12123544)
Supplement: Supplementary file 1 [file cancers-12-03544-s001.pdf]

Hürthle Cells on Fine-Needle Aspiration Cytology are Important for Risk Assessment of Focally PET/CT FDG Avid Thyroid Nodules

David N. Poller, Hakim Megadmi, Matthew J.A. Ward and Pierpaolo Trimboli

Table S1. Malignant Cases: Patient characteristics, PET/CT Findings, SUV*max*, Nodule Size, British Thyroid Association 'U' Category, Thy Cytological Category, Cytological and Histopathological Diagnoses (UICC stage TNM VIII)

Case	Sex	Clinical Characteristics	PET/CT Findings	SUV Max	US Nodule size (mm)	BTA 'U' category	Thy category	Cytology Diagnosis	Histology Diagnosis
1	F	pulmonary nodules & lymph nodes suspicious of metastatic thyroid Ca	Right calcified thyroid gland nodule	2.7	28	4	5	consistent with carcinoma, metastasis cannot be entirely excluded	34 mm follicular thyroid carcinoma, pT2
2	F	squamous cell carcinoma anus, lung & spleen metastases	Right thyroid lobe	9.4	not stated	4	5	in keeping with metastatic anal carcinoma	None, primary anal adeno-carcinoma
3	F	endometrial adeno-carcinoma post radiotherapy	Inferior right thyroid + posterior right thyroid gland	6.1	24 (multiple)	5	5	papillary thyroid carcinoma	15mm papillary thyroid carcinoma, pT1a
4	F	thyroid mass, enlarged lymph nodes cervical levels III & IV	Left thyroid nodule	5.4	not stated infiltrating mass replacing left thyroid lobe	5	4	numerous cells with nuclear pleomorphism and atypia compatible with a neoplasm (non-papillary thyroid	Hodgkin's Lymphoma

								carcinoma or lymphoma)	
5	M	hypercalcemia osteolytic fracture of femur, cystic neck mass	Left thyroid soft tissue posterior margin large cystic nodule	8.9	46	4	1	cystic lesion	carcinoma of parathyroid, cystic with necrotic material
6	F	previous PTC thyroid & endometrial adeno-carcinoma	Left thyroid locally infiltrating	8.7	14 (invades larynx)	not stated	5	pleomorphic nuclei, grooving and chromatin condensation, papillary thyroid carcinoma	none, (cytology showed papillary thyroid carcinoma)
7	M	B cell lymphoma cutaneous nodule arm	Left thyroid	4.1	30	3	3f	small amount of colloid within blood, follicular thyroid neoplasm	40mm follicular variant of papillary thyroid carcinoma, pT3
8	F	adeno-arcinoma colon, lung metastases	Left thyroid	11.0	15	5	5	cytologically malignant-appearing cells in keeping with metastatic adenocarcinoma	none (pancreatic primary adeno-carcinoma)
9	M	squamous cell carcinoma lung	Left thyroid	3.4	12	3	5	metastatic squamous cell carcinoma lung	None (primary squamous cell carcinoma in bronchial biopsy)
10	F	cutaneous malignant	Right thyroid	7.6	16	5	4	suspicious of papillary	22mm multifocal

		malignant melanoma with groin node metastasis						thyroid carcinoma	follicular variant papillary thyroid carcinoma pT2(m)
11	F	malignant melanoma face with regional node involvement	Left thyroid	3.3	6	3	4	suspicious of papillary thyroid carcinoma	6mm papillary thyroid carcinoma pT1a
12	M	adeno-carcinoma oesophagus	Focal nodular uptake	not stated	13mm left & 17 mm right	3	4	suspicious of papillary thyroid carcinoma	none (but clinically papillary thyroid carcinoma not operated)
13	F	previous medullary thyroid carcinoma follow-up	Right thyroid lobe	4.1	47	5	4	ovoid spindled nuclei and pale pink cytoplasm suggestive of medullary carcinoma	45mm medullary thyroid carcinoma, pT3a, pN0
14	F	recent onset left neck mass	Left thyroid bulky partly calcified	10.3	33 & 11	4	3f	follicular thyroid epithelial cells with lymphoid cells without colloid, follicular neoplasm of thyroid	follicular lymphoma, areas up to grade 3B
15	F	papillary thyroid carcinoma on	Soft tissue thyroid bed into tracheal	58.5	not stated tumor invading	5	5	papillary thyroid carcinoma	recurrent papillary thyroid

		long term follow-up	lumen		anterior trachea				carcinoma
16	F	few weeks history of new neck mass	Nodules in both lobes, SUV*max* right 8.2/left 4.1.	8.2	24	not stated	3a	scanty cells exclude neuroendo-crine tumor	medullary thyroid carcinoma
17	F	respiratory symptoms, no tumour elsewhere detected	Right thyroid 2 metabolically active foci	39.0	14	4	3f	neoplastic thyroid cells in sheets	papillary thyroid carcinoma pT1b,(m)
18	M	lung nodules consistent with metastatic thyroid carcinoma	Right Thyroid mass extending into mediastinum	63.2	68	5	3f	sheets of thyroid follicular cells	pT4a follicular thyroid carcinoma, oncocytic, adherent to trachea

Table S2. Hürthle Cell/Oncocytic Cases: Patient characteristics, PET/CT Findings, SUV*max*, Nodule Size, British Thyroid Association 'U' Category, Thy Cytological Category, Cytological and Histopathological Diagnoses.

Case	Sex	Clinical Characteristic s	PET/CT Findings	SUVmax	US Nodule size (mm)	BTA 'U' category	Thy Category	Cytology Diagnosis	Histology Diagnosis
1	F	lymph-adeno pathy & bilateral pleural effusions	right thyroid	3.8	14	2	3f	blood, macrophages & colloid in keeping a cystic thyroid lesion. Many cells oncocytic appearing ? degenerative changes in	Infarcted follicular adenoma

								cyst or oncocytic follicular neoplasm.	
2	F	anal squamous cell carcinoma	right thyroid	30.0	14	4	3f	clustered and single oncocytic cells	follicular adenoma with variable oncocytic change
3	F	anal squamous cell carcinoma	right thyroid	2.9	14	3	3f	follicular neoplasm with oncocytic change	NIFTP with associated oncocytic changes elsewhere
4	F	marginal zone lymphoma parotid	right thyroid	15.1	2 nodules larger 14	3	3f	oncocytic follicular neoplasm	none
5	F	squamous cell carcinoma esophagus	deep left parotid & central thyroid max + patchy uptake left & right thyroid lobes	10.4	12	5	3f	oncocytic cells in keeping with thyroid oncocytic/ Hürthle cell neoplasm or Warthin tumour of salivary gland	none
6	F	adeno-carcinoma lung	right thyroid anterior + left lobe posterior	19.7	40	4	4	oncocytic cells	adenomatoid nodules, focally oncocytic
7	M	renal failure, chronic glomerulo-nephritis	right lobe	5.9	not stated	not stated	3f	blood, rare thyrocytes partly follicular pattern and some cells	none

								with abundant cytoplasm compatible with Hürthle cells	
8	F	inflammatory polyarthritis	left lobe	5.8	9	3	3f	colloid and cells of oncocytic type, an oncocytic follicular neoplasm	refused surgery-surveillance
9	M	diffuse large B cell lymphoma	right lobe	7.3	36	3	3f	thyrocytes with Hürthle cell change, follicular neoplasm with oncocytic change	none
10	F	neuroendo-crine tumour lung	multifocal activity within thyroid	5.5	12	3	3f	blood, bland follicular epithelial cells, some of the cells have oncocytic appearance	none
11	M	malignant mesothelioma	left thyroid	10.6	41	4	3f	oncocytic follicular neoplasm	none
12	F	adeno-carcinoma colon		12.0	19	not stated	3f	oncocytic follicular neoplasm	none
13	F	adenocarcinoma lung	Isthmus (previous hemithyroidectomy)	5.0	14	2	3f	oncocytic cells in second FNA	none

14	M	transient ischaemic attacks exclude autoimmune disease	left thyroid displaces the trachea to the right	20.0	38	3	3f	oncocytic cells	oncocytic follicular adenoma
15	F	colorectal and lung adeno-carcinomas	left lobe	5.0	not stated	3	3a	bland epithelial cells with some colloid with some macrophages and changes which will be in-keeping with cystic degeneration, few oncocytes in second FNA	none

Table S3. Other Cases: Patient characteristics, PET/CT Findings, SUV*max*, Nodule Size, British Thyroid Association 'U' Category, Thy Cytological Category, Cytological and Histopathological Diagnoses.

Case	Sex	Clinical characteristics	PET/CT findings	SUVmax	US nodule size (mm)	BTA 'U' category	Thy category	Cytology Diagnosis	Histology Diagnosis
1	M	squamous cell carcinoma vocal cord	right thyroid gland most likely nodal metastasis	5.6	10	Not stated	1	paucicellular preparation blood and lymphocytes, no colloid and no thyroid follicle epithelial cells	none
2	M	cutaneous malignant melanoma metastatic to stomach	left thyroid	4.6	No definite abnormal nodule identified on US	2	1	cellular aspirate lymphoid cells. No thyroid follicular cells are included and colloid is not a feature so thyroiditis or reactive lymph node	none
3	F	adenocarcin-oma rectum, liver metastasis	right thyroid	5.9	10	4	1	insufficient cells for assessment	none
4	M	adenocarcin-oma rectum	right thyroid	2.2	18	3	1	insufficient cells for assessment but could be a cyst	none
5	F	lung cavitary aspergillosis	right lobe	7.6	14	2	1	colloid fibrinous	none

								material and blood	
6	M	metastatic breast adeno-carcinoma	left thyroid	4.2	20	2	2	blood, some colloid macrophages and small groups of epithelial cells without atypia	benign
7	F	metastatic cutaneous malignant melanoma	left thyroid nodule calcified	5.0	17	4	3f	thyroid follicular cells in keeping with a follicular neoplasm	none
8	F	breast adeno-carcinoma with lung metastases	left thyroid	5.0	11	2	2	benign	none
9	F	endometrial adenocarcinoma	left thyroid	3.9	40	2	1c	Thy1c in 2 aspirates, consistent with a cystic lesion	none
10	F	rheumatoid arthritis	left thyroid	5.3	11	4	3a	series of repeat Thy1's with core biopsy = 'follicular neoplasm' Thy3f equivalent	none
11	F	carcino-sarcoma fallopian tube	right thyroid	4.6	23	3	2	colloid, mixed inflammatory cells, macrophages	hyperplastic nodule

								+ regular follicular cells consistent with benign colloid nodule	
12	F	rheumatoid arthritis	right thyroid	2.9	50	3	3a	low cell yield of bland epithelial cells without atypia but without significant colloid	follicular adenoma
13	F	pulmonary neuroendocrine hyperplasia	left thyroid and also isthmus	3.1	16 and 13 (2 nodules)	2	1	Thy1 aspirates x 2	none
14	F	squamous cell carcinoma lung, right parotid Warthin tumour	left thyroid	4.7	26	2	3a	follicular cells, macrophages and blood without colloid	none
